# Postpartum dysgalactia syndrome in sows: effects on behavior of sows and piglets

**DOI:** 10.1186/s40813-022-00260-3

**Published:** 2022-05-02

**Authors:** Marianne Kaiser, Mette S. Herskin, Stine Jacobsen, Pia H. Andersen, Mai Britt Friis Nielsen, Poul Bækbo, Magdalena Jacobson

**Affiliations:** 1 Department of Animal Science, Aarhus University, 8830 Tjele, Denmark; 2 Department of Veterinary Clinical Sciences, Copenhagen University, 2630 Taastrup, Denmark; 3 Department of Clinical Sciences, Swedish University of Agricultural Sciences, 75007 Uppsala, Sweden; 4 SEGES Innovation P/S, 1709 Axelborg, Copenhagen V, Denmark; 5 Danish Pig Research Centre, SEGES, 8200 Aarhus N, Denmark

**Keywords:** Agalactia, Diagnosis, Inflammation, Milk, Nesting, PDS, Periparturient

## Abstract

**Background:**

To investigate if specific sow and piglet behavioral characteristics could be used diagnostically, this case-cohort study of the behavior of sows and piglets during the periparturient period (from 24 h before the birth of first piglet to 24 h after the birth of last piglet) was conducted. Data included 32 sows diagnosed with PDS (PDS+) vs. 37 healthy matched controls (PDS−) and their litters.

**Results:**

A significant change from active behavior with many postural changes to a more passive behavior was noted in connection with parturition. Sow nesting behavior was less frequent in PDS+ sows compared to PDS− sows during (*p* = 0.04) and after parturition (*p* = 0.0004). No difference was found between PDS+ and PDS− in the number of postural changes, interval from last time standing to the birth of the first piglet, interval from last time eating/drinking to the birth of the first piglet, interval from the birth of the first piglet to the sow standing after farrowing, interval from the birth of the last piglet until eating/drinking, occurrence of bar biting, or frequency or duration of eating/drinking during the observation period. Piglets of PDS+ sows stayed outside the creep areas more often than piglets of PDS− sows (*p* < 0.0001), but there was no difference in the mean number of piglets observed massaging the udder every 10 min.

**Conclusion:**

These results confirm that sow behavior changes from active before parturition to more passive after parturition. Being diagnosed with PDS affected the periparturient behavior of sows as well as their piglets. The observed reduction in nesting behavior in PDS+ sows may suggest that a highly motivated piglet-oriented behavior was affected. However, classical sickness behaviors like lethargy and passivity seem to be poor markers for PDS.

## Background

Postpartum dysgalactia syndrome (PDS) has profound consequences for the welfare of sows [1, 2] and the growth, health and mortality of their piglets [3]. Unfortunately, farmers and veterinarians often experience difficulties in diagnosing the disease. Therefore, a practical diagnostic tool for detecting PDS in sow herds would be advantageous.

We have previously shown that PDS is accompanied by significant changes in inflammatory markers [1, 4] and in increased concentrations of the hormones chromogranin A and cortisol [4]. Similar to other mammals, systemic inflammation in pigs is usually associated with fever [5, 6] as well as behavioral changes such as reduced appetite, lethargy and lack of responsiveness [7, 8]. Thus, since fever and inflammation commonly occur in sows with PDS, behavioral changes indicative of sickness are traditional diagnostic tools that ought to be readily applicable in the detection of PDS. Hence, we hypothesized that recording behaviors indicative of disease during the periparturient period could assist in diagnosing PDS.

Furthermore, studying the behavior of sows in the periparturient period may add to the existing knowledge on PDS. It has been shown that intense nest-building [9] and a higher proportion of sow-initiated suckling bouts [10] are associated with successful nursing (milk let-downs), increased litter size [11] and lower piglet mortality [12]. The behavior of hungry piglets is considered an indicator of milk deficiency in sows and also used in the diagnosis of PDS [13, 14]. However, signs of hunger in the piglets are imprecisely defined in the literature and need to be evaluated in order to add information to our previous findings of reduced growth in litters from PDS− sows [4].

Further, a positive association between the ease of parturition, which has an impact on piglet survival, and the frequency of postural changes during the periparturient period has been demonstrated [15]. Conversely, it has been argued that stress could be a plausible trigger of PDS [16], and it is therefore currently unclear whether *e.g.* restlessness is a positive or negative health indicator. Risk factors for PDS include stressful management and housing [16, 28, 29], and Kulok et al. [17] demonstrated that prenatal stress triggered by maternal confinement caused increased plasma cortisol levels in sows and affected the health of the sows and their piglets. Further, we have previously demonstrated higher concentrations of certain stress markers, such as cortisol, in PDS+ sows before parturition [4]. Despite these indicative results, the assumption that stress is a trigger of PDS remains poorly studied. This study aimed to compare the behavior of PDS-affected (PDS+) and healthy control (PDS−) sows and their piglets during the period from 24 h before the birth of the first piglet to 24 h after the birth of the last piglet.

## Results

### Piglet activity and suckling

Piglets from the PDS+ sows were observed outside the creep areas in a larger proportion of observations than piglets from the PDS− sows (*p* < 0.0001) (Table 1). There was no difference in the number of piglets massaging the udder between litters of PDS+ sows and PDS− sows, neither before (*p* = 0.86) nor after (*p* = 0.12) the sows had received medical treatments (Table 2).

**Table 1:** Percent of observations showing piglets being active outside the creep area by group size and sow status (sows suffering from postpartum dysgalactia (PDS+) and healthy sows (PDS−)). Results are given as a % of all observations

Groups	Group size (No. of Piglets)	Piglets outside the creep area (% of obs.)	*p*-value (all over)
PDS+ sows	0	4	
	1–5	11	
	6–10	13	
	11–15	72	
PDS− sows	0	8	
	1–5	15	
	6–10	16	
	11–15	61	< 0.0001

**Table 2:** Mean numbers of active piglets massaging the udder of sows suffering from postpartum dysgalactia (PDS+) and healthy sows (PDS−) after parturition. There are missing values for 5 sows during the 6 h period after medical treatment

Groups	Active piglets massaging the udder; ‘Nursing’							
	The 6 h preceeding medical treatment of PDS+ sows				The 6 h starting from 24 h after medical treatment of PDS+ sows			
	*n*	Mean (Obs.)	Standard deviation	*p*-value	*n*	Mean (Obs.)	Standard deviation	*p*-value
PDS+	32	6.3	0.5		27	5.9	0.7	
PDS−	33	6.0	0.9	0.86	33	5.6	0.8	0.12

### Sow nesting and bar biting

The occurrence of nest building and bar biting changed over time and these behaviors were more frequently observed before parturition than after (*p* < 0.0001 for both variables) (Table 3). An interaction between time period and treatment group was found with respect to the nest-building behavior (*p* < 0.0001), showing that PDS− sows performed significantly more nest-building behavior than the PDS+ sows during (*p* = 0.04) and after parturition (*p* = 0.0004) (Table 3). In contrast, there was no difference in the occurrence of bar biting between the two groups (*p* = 0.70; Table 3).

**Table 3:** Nesting behavior and bar biting in sows suffering from postpartum dysgalactia (PDS+) and healthy sows (PDS−) during the periparturient period. Results are given as a % of all observations

Sow activity	Relative to parturition	Group	Activity performed	*p*-values	*p*-values for the total period	
			(% of obs.)	(group*)	(group)	(time)
Nesting	Before	PDS+	31			
		PDS−	32	0.56		
	During	PDS+	7			
		PDS−	9	0.04		
	After	PDS+	6			
		PDS−	8	0.0004		< 0.0001
Bar biting	Before	PDS+	23			
		PDS−	23			
	During	PDS+	3			
		PDS−	3			
	After	PDS+	2			
		PDS−	2		0.70	< 0.0001

*Due to the interaction between time period and group, nesting was estimated separately for the time periods before, during and after parturition

### Sow postures

Comparing three time periods (before, during and after parturition), all common postures changed significantly over time (Table 4; *p*-values are given in the table). Active postures were performed more often before parturition compared to after the birth of the piglets. Upright position, mixed recumbency and ventral position, where the belly touched the floor, were performed more frequently during the 24 h before parturition compared to during parturition and the subsequent 24 h period after parturition. Conversely, the sows were positioned in lateral recumbency (right and left) more frequently during and after parturition compared to the 24 h before parturition. Sitting postures occurred more often during parturition compared to before and after. No differences between PDS+ and PDS− sows were found for any kind of active or inactive posture (Table 4).

**Table 4:** Postures in sows suffering from postpartum dysgalactia (PDS+) and healthy sows (PDS−) during the periparturient period

Postures	Group	Before parturition	During parturition*	After parturition	Standard error	*p*-value (time)	*p*-value (group)
Inactive posture, total (h/24 h)	PDS+	16.6	22.6	22.3	–	–	–
	PDS−	16.4	21.8	21.8	–	–	–
Active posture, total (h/24 h)	PDS+	7.3	1.2	1.8			
	PDS−	7.6	1.8	2.2	0.2	< 0.0001	0.06
Ventral recumbency (h/24 h)	PDS+	2.3	0.3	0.7			
	PDS−	2.3	0.4	0.8	0.2	< 0.0001	0.83
Ventral recumbency (% of inactive)	PDS+	14	1	3			
	PDS−	14	2	4	1.2	< 0.0001	0.74
Right or left lateral recumbency (h/24 h)	PDS+	9.5	21.5	20.2			
	PDS−	9.3	20.7	19.6	0.5	< 0.0001	0.25
Right or left lateral recumbency (% of inactive)	PDS+	58	95	91			
	PDS−	56	95	90	1.4	< 0.0001	0.64
Mixed recumbency (Udder partly exposed)	PDS+	28	3	6			
	PDS−	28	2	5	2.7	< 0.0001	0.82
Upright position (h/24 h)	PDS+	4.7	0.8	1.4			
	PDS−	5.1	0.9	1.9	0.2	< 0.0001	0.12
Upright position (% of active)	PDS+	64	46	79			
	PDS−	68	40	82	2.2	< 0.0001	0.89
Sitting (t/24 t)	PDS+	2.7	0.6	0.4			
	PDS−	2.5	1.2	0.4	0.3	< 0.0001	0.26
Sitting (% of active)	PDS+	36	54	21			
	PDS−	32	60	18	3.3	< 0.0001	0.89

Ventral, lateral and mixed recumbency are considered as inactive posture, whereas upright and sitting position are considered as active

*Adjusted to a 24 h period

### Sow behaviors

No differences were observed between the two experimental groups in terms of interval from last time the sows were observed standing and until the birth of the first piglet (*p* = 0.40) or the interval from last observation of eating/drinking to the birth of the first piglet (*p* = 0.20). Likewise, there was no difference between groups for the intervals between the birth of the first piglet and the first occurrence of standing after parturition (*p* = 0.40) or the interval between the birth of the first piglet and the first time sows were observed eating/drinking (*p* = 0.76; Table 5). A median of 7.5 (4.5–11.5) observations of defecation were found for the PDS+ sows during the 24 h pre-parturient period, whereas a median of 0 (0–0) was found during parturition and the 24 h post-parturient period. In the PDS− sows, the corresponding numbers were 7.0 (5–12), 0 (0–0) and 0 (0–0), respectively. Finally, the interval from the birth of the first piglet until the first time the sow was observed urinating did not differ between PDS+ and PDS− sows (*p* = 0.72; Table 5).

**Table 5:** Duration (min) of selected activity performed by sows suffering from postpartum dysgalactia (PDS+) and healthy sows (PDS−) during the periparturient period

Sow activity	Length of time (h:min)				Standard deviation	*p*-value
	PDS+	*n*	PDS−	*n*		
Last standing posture until parturition of first piglet	0:15	32	0:22	37	0:40	0.40
Parturition of the first piglet until first time of standing posture	1:31	32	2:18	37	3:56	0.40
Last time the sow's head was in the trough (eating/drinking) until parturition of first piglet	2:30	32	1:42	37	2:36	0.20
Parturition of the first piglet until first time the sow's head was in the trough (eating/drinking)	8:30	32	8:00	36	1:00	0.76
Parturition of the first piglet until the first time the sow urinated	14:06	29	14:42	29	0:07	0.72

In addition, there were no differences between the groups in the number of times (*p* = 0.94) and the duration of time (*p* = 0.78) spent exhibiting other behaviors (“Others”), or with respect to how many times (*p* = 0.73) and for how long (*p* = 0.31) the sows were observed eating/drinking. On average, PDS+ sows were restrained by a researcher (for sampling, clinical examination and similar purposes) 8.1, 3.6 and 9.7 times before, during and after parturition, respectively. In the PDS− sows, the corresponding number of observations were 8.6, 2.3 and 9.5. Thus, the change over time was significant (*p* < 0.0001), but it applied to both PDS+ and PDS− sows, thus showing no difference between the groups (*p* = 0.91). However, recordings of restraint and treatments by injection were closely correlated and the total time spent restrained did not differ between the groups (*p* = 0.46).

### Delivery of the placenta

In the PDS+ sows, expulsion of the placental tissue was observed 2.6 times on average. The mean interval from the birth of the first piglet until the first placental tissue was observed was 12:49 h (min 9:53; max 15:12). For PDS− sows, a mean of 2.1 observations of expulsion of placental tissue was recorded and the interval from the birth of the first pig until the first observation of the expulsion of placental tissue was 9:04 h (min 7:25; max 11:02) (*p*_*group*_ = 0.01).

## Discussion

### Piglet activity

The high percentage of piglets observed outside the creep areas among the PDS+ sows may indicate that the piglets were hungry, which is in accordance with the lower growth rate compared to the litters from PDS− sows, as previously described in these particular litters [4]. Similarly, other studies indicate that behaviors indicative of hunger as a consequence of PDS manifest as unrest in piglets [13, 14]. The presence of PDS may therefore have welfare implications for the piglets. Whether or not systematic measurements of piglet behavior can be used in the screening of PDS sows requires further investigation, as piglets from healthy PDS− sows also frequently stayed outside the creep area (Table 1). Monitoring piglet behavior may even be considered an inappropriate marker for PDS, since the ambition must be to detect PDS before the piglets show overt signs of hunger. We were not able to show any association between the number of piglets massaging the udder and PDS. This could be due to the fact that both hungry and well-fed newborn piglets are prone to stay by the sow's udder, since the regular cyclical nursing is only gradually established after parturition, as reviewed by Verstegen [18]. In addition, unsuccessful nursing, which makes the piglets massage the udder to a greater extent, is most frequent in the first weeks of lactation [19] and in the same period as the occurrence of PDS.

### Nesting

In the present study, the occurrence of pre-partum nest building did not differ between the two experimental groups, whereas during parturition and post-partum, nest-building behavior was observed significantly more often in the PDS− sows than in the PDS+ sows (Table 3). Typically, nest-building behavior is more common prior to than during or after parturition. In a study of sows kept under semi-natural conditions, nest building was performed 3–7 h before parturition [20]. However, other studies have demonstrated that sows gradually reduce their nesting behavior closer to parturition [21]. According to Damm et al. [22], a nest-building phase peaked on average 7.5 h prepartum, and was followed by nest building during parturition and until 2 h postpartum. In the study by Jensen [20], the sows were restless during parturition, turning around and showing maternal behavior towards the piglets, *e.g.* by grunting and sniffing. Cronin et al. [23] demonstrated sows pawing, rooting and nosing before, during and after parturition, but did not observe pawing and “nesting-like behavior” after parturition. The occurrence of piglet-oriented sow behavior, where sows seem to explore the piglets *e.g.* by sniffing, was also reported by Cronin et al. [23] and Andersen et al. [21]. Finally, Lammers and de Lange [24] showed that gilts with the opportunity to move freely performed a high degree of nest building in the first day after parturition. Thus, healthy sows seem to have a natural preference for nesting or nesting-like behavior both during and after parturition.

The lower level of nesting activity among the PDS+ sows is probably due to sickness, as we have previously shown that these sows had a significantly stronger inflammatory response than the PDS− sows [2]. Passive behavior, *e.g*. lethargy, lower responsiveness and decreased appetite, are well recognized sickness behaviors in both animals and humans [7, 25] and have been observed in sows with parasitic infections [8], systemic inflammation, and fever [5, 26]. However, similar to other studies [23, 24], our results show that most sows were passive after parturition (Table 4), as indicated by increased lying and reduced nest building and bar biting (Table 3). Thus, our results indicate that lethargy, depression and passivity may be poor markers for sickness during the periparturient period of sows, and that disease-related behaviors may be difficult to differentiate from passivity after parturition occurring also in healthy sows. In the case of PDS, terms such as “lethargy” and “lower responsiveness” may therefore need to be replaced by “absence of motivation to nest” and “lack of contact-seeking behavior directed at the piglets”.

“Nesting” may capture a behavior that can distinguish healthy sows from sows with PDS and may partly be used to identify sows at risk of developing PDS. However, further investigations are warranted in this respect. For instance, it may be relevant to examine in detail how pre-parturient nesting behavior differs from intra- and post-parturient behaviors. In this regard, the amount of nesting material and free space available at parturition may be important for the sow’s motivation to perform nesting behavior. Loose-housed sows are more active and exhibit less abnormal behaviors than sows in farrowing crates [27]. In the same study, nesting materials improved sow and piglet investigative behavior. Moreover, it was previously demonstrated that nesting material increased the duration of nest-building and rooting behavior [28] and improved sow-to-piglet communication [10].

### Stress as a cause of PDS

Postpartum maternal characteristics or temperament traits in sows presumably correlate with individual pituitary-adrenocortical stress responses [29] and it has been suggested that stress may be a cause of PDS [16]. Moving sows to the farrowing unit close to expected parturition has therefore been listed as a risk factor for PDS [13] and for mastitis, metritis and agalactia syndrome [30]. The housing system also seems to be a risk factor [30, 31]. We have previously shown that PDS+ sows have higher concentrations of chromogranin A and cortisol compared to PDS− sows [4]. Examples of behavioral indicators of stress in the periparturient period include reduced nest-building behavior [32] or increased bar biting [22, 33]. However, we were unable to detect any difference between PDS+ and PDS− sows in this respect. In addition, since all sows in the present study were exposed to the same environment, it is difficult to argue that PDS+ sows should be more stressed than PDS− sows. However, other studies show that pigs' ability to cope with stress is individual [34–36]. Further, in the study by Malmkvist et al. [37], it was only possible to provoke and detect stress-related aggression and thus reveal individual differences in pigs after exposure to an intruder. Therefore, individual coping strategies could still explain a variation in hormonal and inflammatory concentrations for sows housed in the same environment. Using behavior as the sole method for assessing stress in sows during the periparturient period must therefore still be considered inadequate.

### Placenta

The number of observed placental expulsions may depend on whether the sows expelled one or more placentas at a time or whether this was affected by misinterpretation, where placental tissue was confused with e.g. blood or blood coagulates in the video recordings. Obstetric aid was provided more frequently in the PDS+ sows, as previously reported [4], and this may be the reason for placental remnants occurring slightly more frequently in this group of sows. A longer interval from the birth of the first piglet until an observation of expulsion of placental tissue was observed among the PDS+ sows compared to the PDS− sows, which could be explained by a longer duration of parturition in the PDS+ sows, as previously reported [4]. Finally, delayed placental expulsion in PDS+ sows may be due to weakened labor contractions.

### Defecation

Ceased defecation during parturition and the following 24 h is in good agreement with our previous clinical findings of constipation in both groups of sows over time [38].

## Conclusion

This study documented altered behavior in sows with PDS as well as in their piglets, when compared to healthy matched sows and their litters. However, the behavioral alterations suggest that sickness behavior like lethargy and passivity is difficult to recognize and a poor marker for PDS, as the only difference observed was related to the sows' nesting behavior. Furthermore, these differences could only be detected during and after parturition, and will not meet the desire to identify a behavioral marker for the early detection of PDS. Similar considerations can be made for the behavior of the piglets. Admittedly, significantly more piglets were observed outside the creep areas at PDS+ sows than at PDS− sows, but a large proportion of piglets from both groups stayed outside the creep areas. Moreover, no difference in the number of piglets actively massaging the udder was noted between the groups. Thus, our results indicate that lethargy, depression and passivity may be poor markers for sickness in sows during the periparturient period and that disease-related behaviors may be difficult to recognize.

## Methods

### Animals and housing

The present study was conducted in a Danish commercial sow herd with 600 sows per year. Only Danish crossbred (Landrace/Yorkshire) multiparous sows were included in the study. The herd was enrolled in the Danish Specific Pathogen-Free (SPF) system and further screened for porcine reproductive and respiratory syndrome and influenza prior to data collection. The herd was subjected to a routine animal welfare assessment prior to the start of the study.

From 1 week before expected parturition (approx. gestation day 109) and until weaning, the sows were confined in farrowing crates measuring 1.6 × 2.6 m^2^. The farrowing pens were equipped with covered piglet creep areas with floor heating, rubber mats and dimmable heating lamps (100 W). The pen floor was partly slatted with 2/3 solid concrete at the front and 1/3 iron bars at the back. Between each batch of sows, all farrowing units were cleaned and disinfected. The sows were fed home-mixed liquid feed (50% barley and 50% wheat) four times a day. The sows were restrictively fed (approx. 2.8 kg per day) from 2 days before expected farrowing, according to common Danish practice. After farrowing, the feed ration was increased daily according to the sows' appetite. Sows were given a large handful of cut straw (approx. 10 cm) once a day (in the morning) in accordance with the regulations in the Danish Animal Welfare Act [39]. Prior to the start of the study, the feeding facilities were inspected. Sows and piglets had access to water via one drinking nipple located in the trough (sows) and one attached to the partition in the pen (piglets).

### Study design

The design has previously been described in publications on the inflammatory response [2], hormonal and metabolic changes [4], the prevalence of mastitis, and the clinical alterations pre- and post-partum [38]. In short, a case-cohort study (*n* = 69) was conducted by comparing 32 PDS+ sows to 37 healthy sows (PDS− sows) from 24 h before the birth of the first piglet until 24 h after the birth of the last piglet. All sows were healthy with a fully functional udder at inclusion, but diagnosed as PDS+ if at least two out of the three following characteristics were identified: 1. Reduced feed intake, defined as “trough not empty within 30 min after feeding”, 2. General inflammation of the udder, identified via a subjective assessment of redness, swelling and increased skin temperature, and 3. Rectal temperature ≥ 39.5°C. Pairs of PDS+ and PDS− sows were retrospectively matched based on batch, parity, and time of parturition in the listed order of importance. The litter size was standardized to 15 piglets within 24 h of parturition. Thereafter, cross-fostering or removal of piglets did not take place.

Following the diagnosis of PDS and the subsequent sampling, the PDS+ sows received medical treatment in accordance with the prescriptions made by the herd veterinarian (10,000 IU/kg bw of benzyl procaine penicillin (Noropen® vet., Scan-Vet, Denmark) or 16 mg/kg bw of trimethoprimsulfadiazin (Norodine® vet., ScanVet, Denmark) and 0.4 mg/kg bw of meloxicam (Loxicom®, ScanVet, Denmark). The treatments were administered by the farmer.

### Behavioral observations

The sow and piglet behavior was video recorded (Model IPC-HDW2100P, Dahua Technology Co., Hangzhou, China) using the AxxonNext recording system (AxxonSoft, Skolkovo Innovation Center, Moscow, Russia). The cameras were attached to the ceiling above the rear part of each farrowing pen, allowing a view of the whole pen with the exception of the front part of the trough. The same observer analyzed all video recordings blindly following instruction at start-up and a midway calibration by the project manager.

The behavioral analyses of the video recordings were done by either scan sampling or continuous sampling [40] as well as taking notes of the timing of specific events, such as the birth of the piglets. Definitions of the recorded sow behaviors and other events recorded are listed in Table 6. Continuous sampling was used for the following types of behavior: recumbency (right lateral, left lateral, ventral or mixed), upright, and sitting. For behavioral events, continuous sampling was used throughout the whole study period (from 24 h before the first piglet was born until 24 h after the last piglet was born) for: sows eating or drinking (scoring of which is based on the sow observed with her head in the trough; “Head in the trough”), sow defecation (“Defecation”), sow urinating (“Urination”), other disturbance where a staff member was present in the pen, e.g. to handle piglets ("Disturbance"), when a researcher was handling the sow in the pen, e.g. sampling or performing clinical examinations (“Restraint”), other behavioral events (“Others”), camera dropouts (“Camera”), delivery of placenta (“Placenta”), delivery of a piglet (“Pig”), “Obstetric aid” performed by staff member, and any treatment given by injection (“Treatment”). In addition, the occurrence of nest-building behavior (“Nesting”) and sows biting the farrowing rail (“Bar biting”) was recorded throughout the whole study period by One-zero sampling in 5 min intervals [40] (Table 6).

**Table 6:** Definition of selected behavioral states and events in sows from 24 h before parturition of the first piglet until 24 h after parturition of the last piglet, based on two studies [4, 41]

States	Definitions	Observation technique
Right or left lateral recumbency	Right or left side of the head, right or left scapula and waist touch the floor and the udder is visible [slightly adjusted according to Cui et al. [41]]	Continuously
Mixed recumbency	Lying with the udder partly exposed where only some parts of the udder are visible	Continuously
Ventral recumbency	Stomach touches the floor, the front legs are stretched or folded under the body [slightly adjusted according to Cui et al. [41]]	Continuously
Upright position	Four legs are placed on the floor or moving—without changing the position of the body or leading to a horizontal movement of the body	Continuously
Sitting	Sitting like a dog, hind quarter and caudal end touching the floor with hind legs folded and front legs erect to support the body weight (slightly adjusted according to Cui et al. [41])	Continuously
*Events*		
Head in the trough*	Head (from the ears onward) in the trough. Ends when the sow removes her head from trough	Continuously
Defecating	Sow defecating	Continuously
Urination	Sow urinating	Continuously
Disturbance	A staff member handling the sow in the pen (handling piglets, emptying the trough, etc.)	Continuously
Restraint	A researcher handling the sow in the pen (sampling, performing clinical examination, feeding sucker, forcing the sow to stand up, etc.)	Continuously—from 10 min. before handling to 10 min. after
Others	The sow shows other behavioral events than described	Continuously
Camera	The camera prevents a view of the sow (including power failure)	Continuously
Nesting	Sitting or standing. Pawing with a foreleg, rubbing their head or rooting or biting with the snout against the farrowing rail	One-zero sampling in 5 min intervals
Bar biting	Biting with the snout against the farrowing rail due to stereotyped behavior	One-zero sampling in 5 min intervals
Pig	A piglet is delivered	Continuously
Placenta	The placenta is delivered	Continuously
Obstetric aid	A staff member provides obstetric aid (results reported in Kaiser et al. [2])	Continuously
Treatment	Injection treatment of sick sows	Continuously

*Indicates an approximate (‘most likely’) measure of water and feed intake

**Fig. 1:**
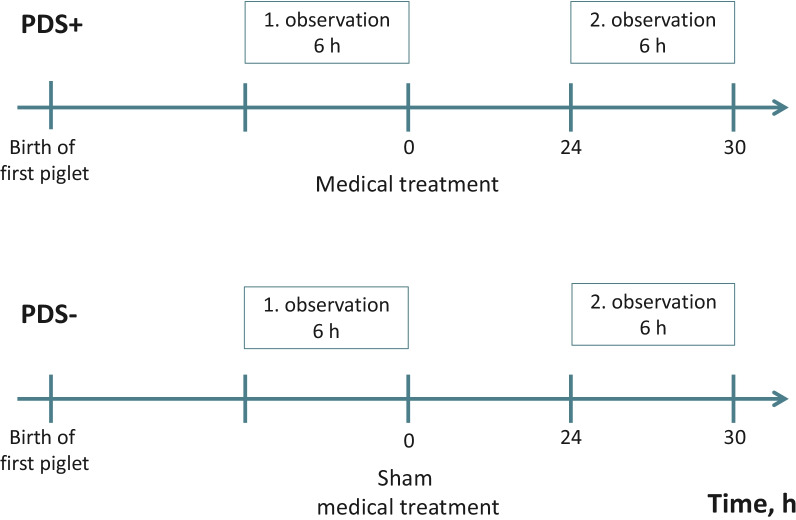
Two observation periods of non-sleeping piglet activities from sows suffering from post partum dysgalactia syndrome (PDS+) and healthy sows (PDS−). The first observation was performed 6 h before the medical treatment of PDS+ sows (which occurred between 6 h and 26.9 h after parturition) and the second observation was performed 24–30 h after the medical treatment. Based on matching with PDS+ sows, equivalent fictitious treatment times and observation periods were determined for the healthy PDS−sows

For two defined time periods within the total observation period, scan sampling [40] was used to observe piglet behavior. Thus, the number of piglets located outside the creep area was scanned every 5 min (“Creep area”), and the number of piglets massaging the udder was scanned every 10 min (“Suckling”) (Table 7). The number of piglets located outside the creep area was pooled into four different categories: 0, 1–5, 6–10 or 11–15 piglets. As illustrated in Fig. 1, these two variables were observed during two periods where we expected that PDS− sows would provide less milk: (1) during the six hours preceding the medical treatment of the PDS+ sows, and (2) six hours starting from 24 h after the medical treatment. In the untreated PDS− sows, a sham “medical treatment time” and the associated observation periods were defined, corresponding to the observations made in the matched PDS+ sows. For example, if a PDS+ sow was treated medically eight hours after the birth of the first piglet, the “treatment time” for the corresponding PDS− sow would also be eight hours after the birth of the first piglet. In case of missing values due to technical difficulties, the fictive “medical treatment time” was defined as the average time of treatment for all medically treated sows (which occurred 18:35 h after the first piglet was born) (Fig. 1).

**Table 7:** Definition of selected piglet behaviors

Events	Definitions of piglet activity	Observation technique
Creep area*	Number of piglets outside the covered creep areas	Scanning every 5 min
Nursing	Number of active (non-sleeping) piglets massaging the udder	Scanning every 10 min

*Piglets were grouped into 4 different categories of 0, 1–5, 6–10 or 11–15 piglets

### Statistical analysis

All statistical analyses were performed according to procedures described in the Statistic Analytical Software Enterprise Guide 7.1 (SAS Institute, Cary, NC). Not all periods had similar durations due to farrowing times and because some video recordings were missing. The time spent in the different recumbences (right lateral, left lateral, mixed and ventral recumbency as well as sitting position) were analyzed statistically as continuous variables in a linear mixed model with sow as a random effect in the PROC MIXED procedure. The frequency of defecation, urination, disturbance, placenta, obstetric aid, nesting, bar biting, creep area, suckling, feeding/drinking, restraint, and camera, as well as the time of delivery of each piglet were analyzed using the PROC GLIMMIX procedure, with sow as a random effect. The variables on frequency of behaviors were assumed to be Poisson or Binomial distributed. The number of defecations was calculated descriptively for the sows for each period and given as a median value (25th and 75th percentile). The difference in interval from the birth of the first piglet until the first placenta was observed between the two groups of sows was tested by a simple t-test in the PROC TTEST procedure. For observations that only occurred once per sow (medical treatment), non-parametric Wilcoxon rank tests were used for estimating in PROC NPAR1WAY. Due to occasional power outages in the barn, observations were not recorded from one sow after parturition.

## Data Availability

The corresponding author can provide access to the datasets upon request.

## Abbreviations

PDS: Postpartum dysgalactia syndrome (PDS+ is cases and PDS− is healthy sows)
SPF: Specific Pathogen-Free
